# Endovascular Management of a Giant Supraclinoid Internal Carotid Artery Dissecting Pseudoaneurysm Using Flow Diversion and Coiling: A Case Report and Review of Literature

**DOI:** 10.1002/ccr3.72967

**Published:** 2026-06-22

**Authors:** M. Kodeeswaran, S. P. Goutham, M. Naveenkumar, K. P. Priyadarshan, Moyniul Haq, Anand Warrier, Rimpy Jospeh, Shakir Husain Hakim, Bipin Chaurasia

**Affiliations:** ^1^ Department of Neurosurgery Government Kilpauk Medical College and Hospital Chennai Tamil Nadu India; ^2^ VIMS Hospitals Salem Tamil Nadu India; ^3^ Neurosurgery Academy and Research Foundation/Department of Neurosurgery, Government Kilpauk Medical College and Hospital Chennai Tamil Nadu India; ^4^ Prashanth Hospitals Chennai Tamil Nadu India; ^5^ EMS Memorial Cooperative Hospital and Research Centre Perinthalmanna Kerala India; ^6^ Baby Memorial Hospital Calicut India; ^7^ Department of Neurosurgery Neurosurgery Clinic Birgunj Nepal

**Keywords:** coiling, dissecting aneurysm, endovascular treatment, flow diversion, intracranial pseudoaneurysm, supraclinoid ICA

## Abstract

We present a case of a giant left supraclinoid ICA dissecting pseudoaneurysm managed successfully with flow diversion and adjunctive coiling, highlighting radiological findings and clinical outcome. Endovascular intervention provides a minimally invasive and durable solution with good clinical and radiological outcomes.

## Key Clinical Massage

Endovascular intervention provides a minimally invasive and durable solution with good clinical and radiological outcomes for a case of a giant left supraclinoid ICA dissecting pseudoaneurysm. We highlight the role of flow diversion with adjunctive coiling as a safe and durable treatment strategy in anatomically challenging intracranial pseudoaneurysms.

## 1 Introduction

Dissecting pseudoaneurysms of the intracranial internal carotid artery (ICA) are exceedingly rare and represent a distinct subset of vascular lesions characterized by disruption of the arterial wall layers, leading to the formation of a false lumen. These lesions are particularly prone to rupture and thromboembolic events, and their presentation is often insidious, with symptoms ranging from headache and visual disturbances to cranial neuropathies or transient ischemic attacks [1, 2]. The supraclinoid segment of the ICA poses additional treatment challenges due to its deep location, close association with critical neurovascular structures, and limited surgical access.

Conventional microsurgical approaches, while effective in select cases, carry considerable morbidity, especially when dealing with large or giant pseudoaneurysms. Endovascular techniques have emerged as the preferred modality for managing such lesions, offering reduced invasiveness and improved access to complex aneurysmal anatomy [3]. Among these, flow‐diverting stents have revolutionized the treatment landscape by promoting endothelialization and parent vessel reconstruction, thereby allowing for gradual exclusion of the aneurysm from the circulation [4, 5]. When combined with adjunctive coiling, especially in cases with mass effect or a high risk of rupture, this approach enhances immediate aneurysm stability while enabling long‐term remodeling.

We present a rare case of a giant dissecting pseudoaneurysm arising from the supraclinoid segment of the left ICA in a 48‐year‐old female, managed successfully with endovascular flow diversion and adjunctive coiling. This case underscores the efficacy of modern neurointerventional strategies in the management of anatomically complex and high‐risk intracranial vascular lesions.

## 2 Case History/Examination

A 48‐year‐old right‐handed female presented with a two‐month history of persistent, dull, left‐sided headache that was non‐throbbing in nature and unresponsive to over‐the‐counter analgesics. In the week prior to presentation, she developed intermittent visual blurring along with recent onset of right upper limb weakness. There was no history of trauma, seizures, or prior neurological illness.

On neurological examination, she exhibited right upper limb pyramidal signs, including increased muscle tone, brisk deep tendon reflexes, and mild weakness (Medical Research Council grade 4+/5). Cranial nerve examination was normal, and no sensory deficits or signs of raised intracranial pressure were noted.

## 3 Methods (Differential Diagnosis, Investigations, and Treatment)

**FIGURE 1:**
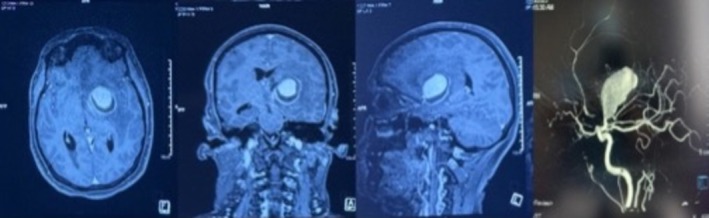
MRI brain (T2‐weighted axial image) showing a large flow void in the supraclinoid segment of the left ICA with perilesional gliotic changes in the left ganglio‐capsular and corona radiata regions. Mild rightward midline shift is noted.

Magnetic resonance imaging (MRI) of the brain revealed a large flow void in the supraclinoid region of the left internal carotid artery (ICA), associated with surrounding mass effect and mild midline shift to the right. There were gliotic changes noted in the left ganglio‐capsular region, corona radiata, and centrum semiovale, without evidence of acute infarction or hemorrhage. MR angiography confirmed the presence of a giant dissecting pseudoaneurysm arising from the supraclinoid segment of the left ICA (Figure 1). Additional vascular findings included a hypoplastic left A1 segment and a fetal origin of the right posterior cerebral artery, with preserved flow in the remaining intracranial vasculature.

**FIGURE 2:**
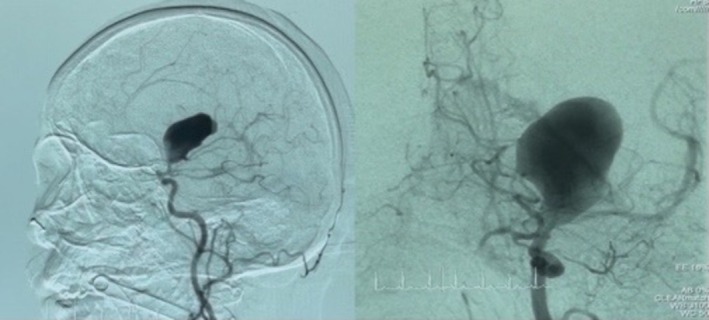
3D digital subtraction angiography reconstruction demonstrating a giant dissecting pseudoaneurysm arising from the supraclinoid segment of the left ICA.

In view of the aneurysm's size, critical location, and symptomatic progression, the patient was planned for endovascular intervention. The patient received dual antiplatelet therapy (aspirin and clopidogrel) for 5 days prior to the intervention. Under general anesthesia, right femoral arterial access was obtained using the Seldinger technique, and a 6F guiding catheter was advanced into the left ICA under fluoroscopic guidance in a biplanar neuroangiography suite. Digital subtraction angiography (DSA) confirmed a dissecting pseudoaneurysm measuring approximately 12 mm, with preserved distal flow (Figure 2).

**FIGURE 3:**
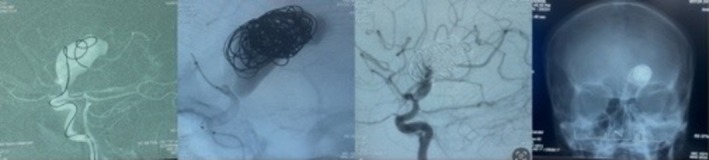
Intraoperative fluoroscopic and post‐procedure angiographic images showing coil deployment within the aneurysm sac and near‐complete exclusion of the pseudoaneurysm with preserved distal ICA and MCA flow.

A flow‐diverting stent Pipeline Embolization Device (Medtronic) was deployed across the diseased segment, extending from the supraclinoid ICA to the proximal M1 segment of the middle cerebral artery. Adjunctive embolization of the aneurysm sac was performed using multiple soft platinum detachable coils, achieving dense packing to promote rapid thrombosis and reduce intra‐aneurysmal flow (Figure 3). Intraoperatively, systemic heparinization was administered to maintain an activated clotting time (ACT) above 250 s. Continuous neurophysiological monitoring was employed throughout the procedure. Final angiography demonstrated near‐complete exclusion of the aneurysm with preserved patency of the parent artery and distal branches.

Following the procedure, the patient was extubated and transferred to the neurointensive care unit for observation. Postoperative management included continuation of dual antiplatelet therapy. Her clinical course was uneventful, with gradual improvement in right upper limb strength. By the second postoperative week, motor function had returned to near‐normal levels.

## 4 Conclusion and Results (Outcome and Follow‐Up)

**FIGURE 4:**
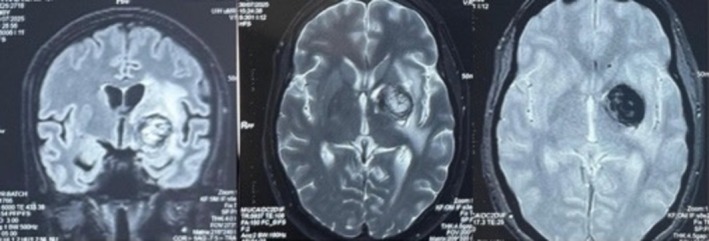
MRI (SWI sequence) at 3‐month follow‐up showing blooming artifact due to coils in the basal ganglia region with no new infarcts or hemorrhage.

On follow‐up MRI performed 3 months later, the aneurysm remained excluded with no evidence of new infarction or hemorrhage. The flow‐diverting stent was well‐positioned, and susceptibility artifacts from the coils were evident in the left basal ganglia (Figure 4). Persistent gliotic changes and a mild midline shift were noted, consistent with the preexisting mass effect. The patient remained neurologically stable with no new deficits and demonstrated significant clinical improvement.

## 5 Discussion

Dissecting pseudoaneurysms of the intracranial internal carotid artery (ICA), particularly within the supraclinoid segment, are uncommon and often pose both diagnostic and therapeutic challenges. These lesions involve an intimal tear that allows blood to enter the arterial wall, forming a false lumen. The supraclinoid ICA, due to its structural characteristics including the absence of an external elastic lamina and a relatively thin tunica media, is especially vulnerable to dissection and aneurysm formation. Clinical presentations are variable and may include headache, visual disturbances, ischemic events, or subarachnoid hemorrhage, depending on the lesion's size, location, and hemodynamic stress [6, 7].

In the present case, the patient exhibited left‐sided headache, transient visual impairment, and motor weakness, correlating with the mass effect and gliotic changes observed on neuroimaging. MRI and MRA revealed a large dissecting pseudoaneurysm in the left supraclinoid ICA, along with ischemic gliosis in the left ganglio‐capsular and corona radiata regions, likely reflecting chronic vascular insufficiency. The lesion's size and symptomatic profile necessitated urgent and definitive management.

Open surgical approaches such as clipping, trapping, or bypass are associated with high technical difficulty and morbidity due to the deep and complex anatomy of the supraclinoid ICA. Recent advancements in endovascular therapy, particularly the use of flow‐diverting stents such as the Pipeline Embolization Device (PED), have redefined treatment paradigms. Flow diverters offer a minimally invasive alternative that promotes aneurysm thrombosis, preserves flow through the parent vessel, and encourages vascular remodeling [8, 9].

In large, symptomatic, or high‐risk pseudoaneurysms, adjunctive coiling can be used alongside flow diversion to promote early thrombosis and reduce the risk of delayed rupture, which may occur during the endothelialization phase [10]. This combined strategy is associated with faster symptom resolution, improved occlusion rates, and decreased retreatment requirements [11].

In this case, a PED was deployed from the supraclinoid ICA to the M1 segment of the left middle cerebral artery, along with dense coiling of the aneurysmal sac. Post‐procedural angiography confirmed near‐complete aneurysm exclusion with preserved distal flow. Follow‐up MRI showed no new infarctions or hemorrhages, and the patient had marked neurological recovery.

Anatomical variations in the Circle of Willis, such as the hypoplastic left A1 segment and fetal origin of the right posterior cerebral artery observed in this case, highlight the importance of detailed preoperative vascular mapping. These variations can influence perfusion dynamics and procedural planning, underlining the need for individualized treatment strategies [12].

Our findings are consistent with the systematic review and meta‐analysis by Duan et al., which included 548 cases of intracranial dissecting aneurysms treated with flow diverters. The review demonstrated an 84.2% complete occlusion rate and favorable clinical outcomes in 90.4% of patients, supporting the safety and efficacy of flow diversion for these complex lesions [13].

Similarly, Chen et al. compared flow diversion with stent‐assisted coiling (SAC) and found that although both methods are effective, flow diversion offers higher long‐term occlusion rates and a reconstructive advantage. Despite a slightly increased risk of periprocedural ischemia, flow diverters showed superiority in preserving parent vessel patency and remodeling fusiform or wide‐necked aneurysms [14]. Our patient's excellent outcome supports these findings and demonstrates the utility of adjunctive coiling in reducing early rupture risk and expediting symptom resolution.

Aydin et al. reported a 92.6% aneurysm occlusion rate with minimal morbidity in patients with supraclinoid ICA aneurysms treated primarily with PED in a multicentre Turkish study. The favorable anatomical characteristics of the supraclinoid segment, including vessel geometry and collateral flow, contribute to the success of flow diversion in this location [15, 16, 17, 18]. Our patient's course mirrored these results, reinforcing the efficacy of this treatment approach.

Additionally, Kumar et al. from India reported their experience using stent‐assisted coiling and covered stents in ICA pseudoaneurysms. While their outcomes were favorable, the complication rate was higher (10%) compared to studies using flow diversion [16]. This supports the growing trend toward reconstructive, durable approaches such as flow diversion, especially for complex or dissecting lesions.

Table 1 summarizes key studies that have influenced the management of MCA and supraclinoid ICA aneurysms, detailing treatment modalities and associated outcomes. These include both surgical and endovascular strategies, reflecting the shift toward less invasive yet effective interventions in complex aneurysm cases.

**TABLE 1:** Summary of key literature on dissecting or pseudoaneurysms of the supraclinoid ICA and related intracranial segments, highlighting evolving treatment strategies and outcomes.

Author	Aneurysm type/site	Treatment modality	Outcome/Implication
Malek et al. [6]	Dissecting supraclinoid ICA	Pathophysiological insight	Described vulnerability due to structural weakness of supraclinoid ICA
Debette & Leys [7]	Intracranial arterial dissection	Clinical and imaging correlation	Highlighted variable presentations and importance of MRI/MRA in diagnosis
Brinjikji et al. [11]	Pipeline‐treated aneurysms	Meta‐analysis	Reported high occlusion and low retreatment with PED; noted ischemic risk in select cases
Kumar et al. [16]	ICA pseudoaneurysms (Indian cohort)	Covered stents/SAC	Effective, but noted higher complication rates; highlighted shift toward flow diversion
Dmytriw et al. [8]	Complex intracranial aneurysms	Flow diverters	Emphasized reconstructive benefits of flow diversion in complex morphologies
Martínez‐Galdámez et al. [9]	Intracranial aneurysms	Multicenter experience with PED	Confirmed durability and remodeling benefits of flow diverters in intracranial circulation
Aydin et al. [15]	Supraclinoid ICA aneurysms	Turkish multicenter PED study	Achieved 92.6% occlusion with low morbidity; supports flow diversion in this location
Cagnazzo et al. [10]	Large or symptomatic aneurysms	Flow diversion ± coiling	Reported improved occlusion and safety with adjunctive coiling in select cases
Duan et al. [13]	Intracranial dissecting aneurysms	Systematic review (PED cases)	Found 84.2% occlusion rate and 90.4% favorable outcomes with flow diversion
Chen et al. [14]	Dissecting/fusiform aneurysms	PED vs. stent‐assisted coiling	Flow diversion superior in long‐term occlusion; SAC still effective in certain cases

This case contributes to the growing body of literature supporting the use of flow‐diverting stents, either alone or in combination with adjunctive coiling, as an effective treatment strategy for dissecting pseudoaneurysms of the supraclinoid internal carotid artery. The successful aneurysm exclusion, parent artery preservation, and full neurological recovery seen in this patient affirm the safety and effectiveness of this minimally invasive strategy. Long‐term imaging follow‐up remains essential to confirm sustained occlusion and arterial remodeling.

## 6 Conclusion

This case demonstrates the efficacy and safety of a combined endovascular approach using flow diversion and adjunctive coiling in the treatment of a giant, unruptured dissecting pseudoaneurysm of the supraclinoid internal carotid artery. The flow‐diverting stent facilitated durable aneurysm exclusion and parent vessel reconstruction, while adjunctive coiling enhanced immediate intra‐aneurysmal thrombosis, reducing the risk of delayed rupture. The patient's favorable neurological recovery without periprocedural complications underscores the value of this minimally invasive strategy. These findings are consistent with both international and regional data supporting flow diversion as a preferred modality in complex intracranial pseudoaneurysms. Timely diagnosis, tailored procedural planning, and vigilant follow‐up imaging remain key to optimizing patient outcomes in such challenging cerebrovascular lesions.

## Author Contributions

**M. Naveenkumar:** investigation, methodology. **S. P. Goutham:** methodology, software. **Shakir Husain Hakim:** methodology, visualization, supervision. **K. P. Priyadarshan:** software, formal analysis. **Anand Warrier:** data curation, formal analysis, visualization. **Bipin Chaurasia:** writing – review and editing, visualization, validation, supervision. **M. Kodeeswaran:** conceptualization, investigation, writing – original draft, methodology. **Moyniul Haq:** data curation, supervision, resources. **Rimpy Jospeh:** methodology, validation, visualization.

## Funding

The authors have nothing to report.

## Ethics Statement

The study was conducted in accordance with institutional ethical guidelines and the Declaration of Helsinki.

## Consent

Informed written consent was taken from patient prior to initiation of the project.

## Data Availability

The authors have nothing to report.
